# Genetic diversity assessment of *Helichrysum arenarium* (Asteraceae) for the genetic restoration of declining populations

**DOI:** 10.1002/ece3.10953

**Published:** 2024-02-15

**Authors:** Fabienne Van Rossum, Cécile Godé, Alenka Baruca Arbeiter, Olivier Raspé, Melike Simsek, Benjamin Barigand, Olivier J. Hardy, Dunja Bandelj

**Affiliations:** ^1^ Meise Botanic Garden Meise Belgium; ^2^ Service Général de l'Enseignement Supérieur et de la Recherche Scientifique, Fédération Wallonie‐Bruxelles Brussels Belgium; ^3^ Univ. Lille, CNRS, UMR 8198 ‐ Evo‐Eco‐Paleo Lille France; ^4^ Faculty of Mathematics, Natural Sciences and Information Technologies University of Primorska Koper Slovenia; ^5^ Unit of Evolutionary Biology and Ecology Université Libre de Bruxelles Brussels Belgium

**Keywords:** endangered species, geitonogamous selfing, genetic restoration, *Helichrysum arenarium* (L). Moench, plant translocations

## Abstract

*Helichrysum arenarium* (L.) Moench (Asteraceae) is a self‐compatible, insect‐pollinated herb occurring in sand grasslands, and is declining and endangered in many parts of its European distribution range. A recovery plan of *H. arenarium* has been conducted in southern Belgium, involving plant translocations. We developed multiplex genotyping protocol for nine microsatellite markers previously published for *Helichrysum italicum* and two newly developed microsatellite markers for *H. arenarium*. Eleven polymorphic loci were associated (pooled) in two multiplex panels, to assess the genetic status of the only small remaining population in Belgium and of three large German populations used as seed source for propagating transplants. The small Belgian population was characterized by high clonality, with only two, however heterozygous, genets detected. The three large German populations showed high genetic diversity (*H*_e_ ranging from 0.635 to 0.670) and no significant inbreeding coefficient values, despite expectations of geitonogamous selfing. Management practices (grazing livestock) increasing seed dispersal distances, inbreeding depression at early stages of development, and mechanisms preventing or delaying selfing might be hypothesized to explain the observed patterns. The two Belgian genotypes remained within genetic variation range of German populations so that the high genetic differentiation between Belgian and German populations (*F*_ST_ values ranging from 0.186 to 0.206) likely resulted from genetic drift effects and small sample size. Transplants obtained from seeds sampled from the three large source populations from Germany constitute a highly diverse, noninbred gene pool, and are thus of high genetic quality for plant translocations.

## Graphical abstract

*Helichrysum arenarium* (Asteraceae) is a self‐compatible, insect‐pollinated herb from sand grasslands, and is declining and (critically) endangered in Europe. Genetic assessment using microsatellite markers of the only small remaining population in Belgium and of three large German populations revealed high clonality, with only two, however heterozygous, genets detected in the small Belgian population, and high genetic diversity and no significant inbreeding coefficient values, despite expectations of geitonogamous selfing, in the three large German populations, which will be used for plant translocations.

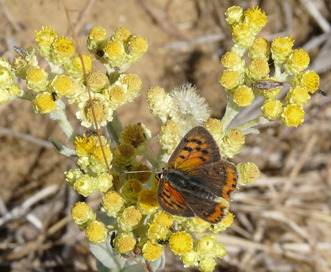

## 1 INTRODUCTION

Assessment of genetic diversity of populations of critically endangered species has been recognized as important for the preparation of restoration projects, and genetic restoration as an essential goal in conservation achievements (e.g., Broadhurst et al., 2023; De Vitis et al., 2022; Mijangos et al., 2015; Wei et al., 2023). Indeed, genetic tools allow for identifying a number of key processes involved in population viability, in particular for plant species: mating processes, genetic erosion, inbreeding, pollen and seed dispersal, sexual recruitment, clonal extent, and progeny genetic quality (Aguilar et al., 2019; Doyle et al., 2023; Van Rossum, 2023; Van Rossum et al., 2022). Depending on the identified failures, additional interventions to ecological restoration may be required to achieve demographically and genetically viable and evolutionary resilient populations (De Vitis et al., 2022; Gargiulo et al., 2021; Ottewell et al., 2016). For instance, increasing genetic diversity and the number of compatible mates is necessary when spatially isolated plant populations are genetically depauperate and inbred, which may be achieved by assisted gene flow actions such as hay transfer, plant translocation, and cross‐pollination (e.g., Barmentlo et al., 2018; Kaulfuß & Reisch, 2021; Ottewell et al., 2016; Ralls et al., 2018). Accordingly, the development of polymorphic molecular markers, preferably codominant, to estimate inbreeding, such as microsatellites (simple sequence repeats, SSRs) or single nucleotide polymorphisms (SNPs), is required to quantify genetic diversity and structure parameters that will be used to assess population genetic status (e.g., Williams et al., 2014).

*Helichrysum arenarium* (L.) Moench, an Everlasting species (Asteraceae), typically occurs in pioneer vegetation stage of dry sand grasslands, and is widely spread from eastern Europe to Asia, reaching its western margin in southern Belgium, Luxembourg, and eastern France (Pljevljakušić et al., 2018). In many countries, *H. arenarium* is protected because it is declining and endangered not only as a result of destruction and fragmentation of its habitats but also due to inflorescence picking for pharmaceutical and ornamental (dry‐flower decorations) use (Dănăilă‐Guidea et al., 2022; Godefroid et al., 2016; Parent, 1986). In Belgium, *H. arenarium* was formerly widely distributed in the Lorraine phytogeographic district (southern Belgium) but has strongly declined due to habitat destruction resulting from exploitation of sand quarries (however also temporarily providing secondary habitats), urbanization, and conifer plantations, and due to spontaneous recolonization by competing vegetation, shrubs, and forest (Parent, 1986). In 2013, only one natural population remained, consisting of one patch of about 30 rosettes along a cliff (Godefroid et al., 2016). Therefore, along with the ecological restoration of dry sand grassland areas in southern Belgium, it has been decided to conduct plant translocations for population reinforcement and reintroductions, using nonlocal seed sources. The selected sources were the ecologically and geographically closest large populations, located in North Rhine‐Westphalia (western Germany) (Godefroid et al., 2016).

In this study, we demonstrated the transferability of microsatellites designed for *Helichrysum italicum* (Roth) G. Don (Baruca Arbeiter et al., 2021) to the commercially important *H. arenarium*, and we developed two new microsatellite markers. A rapid, multiplex genotyping protocol for *H. arenarium* was developed, involving 11 microsatellite loci. We used these polymorphic markers to characterize genetic diversity and structure of the only remaining Belgian population and of the German seed source populations used for translocation. In particular, we estimated genetic diversity, inbreeding levels and clonal extent within populations, and genetic differentiation among populations, and discussed our findings in relation to species life‐history traits (e.g., clonal propagation ability, self‐compatible breeding system, insect pollination, and anemochory) and site management. More specifically, we addressed the following questions: (1) What is the genetic status of the last relictual Belgian population in terms of genetic diversity and clonal extent? (2) Is genetic diversity of the German source populations high enough but not too distinct from the only remaining Belgian population to use them for reinforcing the later? (3) Is there evidence of inbreeding due to (geitonogamous) selfing or mating between relatives that could slow down genetic mixing between sources in the translocated populations?

## 2 MATERIALS AND METHODS

### 2.1 Study species and populations

**FIGURE 1:**
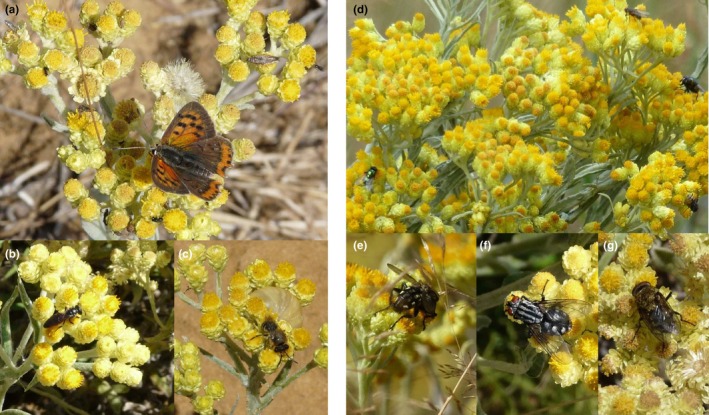
Flowering inflorescences of *Helichrysum arenarium* with visiting insects in Belgian Lorraine. (a) *Lycaena phlaeas* and a microlepidoptera (Lepidoptera) and *Chalcidoidea* wasps (Hymenoptera); (b) *Sphecodes* sp. (Hymenoptera); (c) *Colletes* sp. (Hymenoptera); (d–g) various flies (Diptera), with (e) *Sarcophaga carnaria*; (pictures a–c, f–g F. Van Rossum; pictures d–e D.J. Parmentier).

**FIGURE 2:**
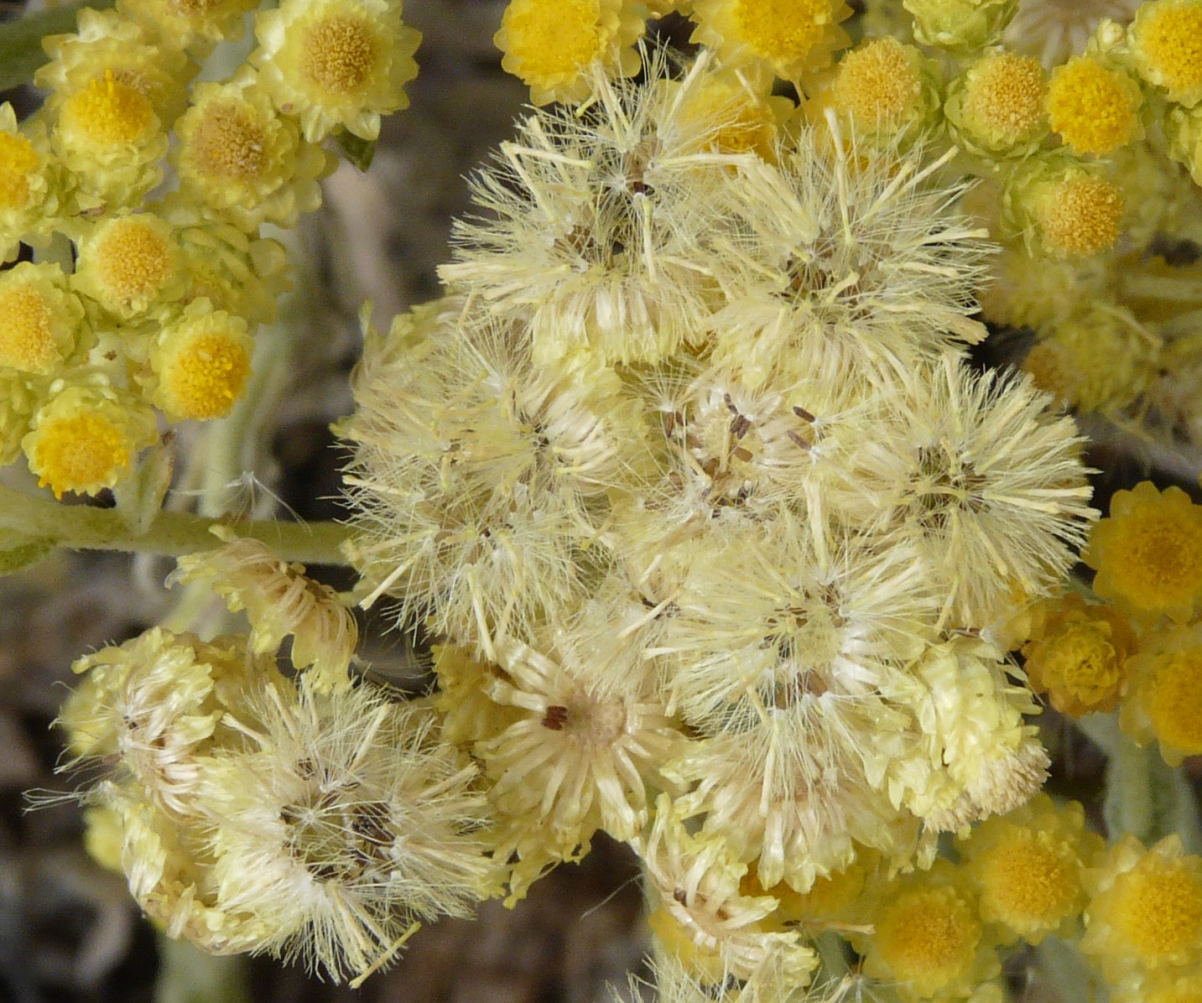
Fruiting flower heads just before seed dispersal (achenes with their pappus) (picture: F. Van Rossum).

*Helichrysum arenarium* is a self‐compatible, insect‐pollinated, rosette‐forming herb species, which clonally propagates by rhizomes (Klimešová & Klimeš, 2013; Klotz et al., 2002). Fully grown rosettes reach 4–12 cm diameter (Godefroid et al., 2016; Pljevljakušić et al., 2018). Flowering occurs from July to October. Flowering stalks of 10–50 cm high bear numerous small bright yellow to orange flower heads (capitula) of 3–6 mm diameter, grouped in false umbels. Florets are usually hermaphroditic and homogamous, but can be female at the outer whorl of the capitulum, and produce nectar hidden within the corolla tube (Godefroid et al., 2016; Klotz et al., 2002; Knuth, 1908; Pljevljakušić et al., 2018). Capitulum flowering starts from the outer to the inner floret whorls (Figure 1b). Flowers of *H. arenarium* are important resources for pollinators, which can be wild bees, bumblebees, wasps, hoverflies, flies, Bombyliidae, and butterflies (Beil et al., 2008, 2014; Klotz et al., 2002; Kratochwil et al., 2009; F. Van Rossum, pers. obs.; Figure 1). The fruit is a small achene of 0.7–1.2 mm long, with a hairy pappus allowing for anemochory (Klotz et al., 2002; Pljevljakušić et al., 2018; Figure 2). The species, especially its flower heads, has recognized medicinal properties (e.g., antimicrobial, antioxidant, and anti‐inflammatory) and also contains chemical compounds useful for cosmetic and food industry applications (Dănăilă‐Guidea et al., 2022; Kramberger et al., 2021; Pljevljakušić et al., 2018). Flower stalks have also been popular for ornamental dry‐bouquet arrangements and as moth repellent (Dănăilă‐Guidea et al., 2022; Parent, 1986).

**FIGURE 3:**
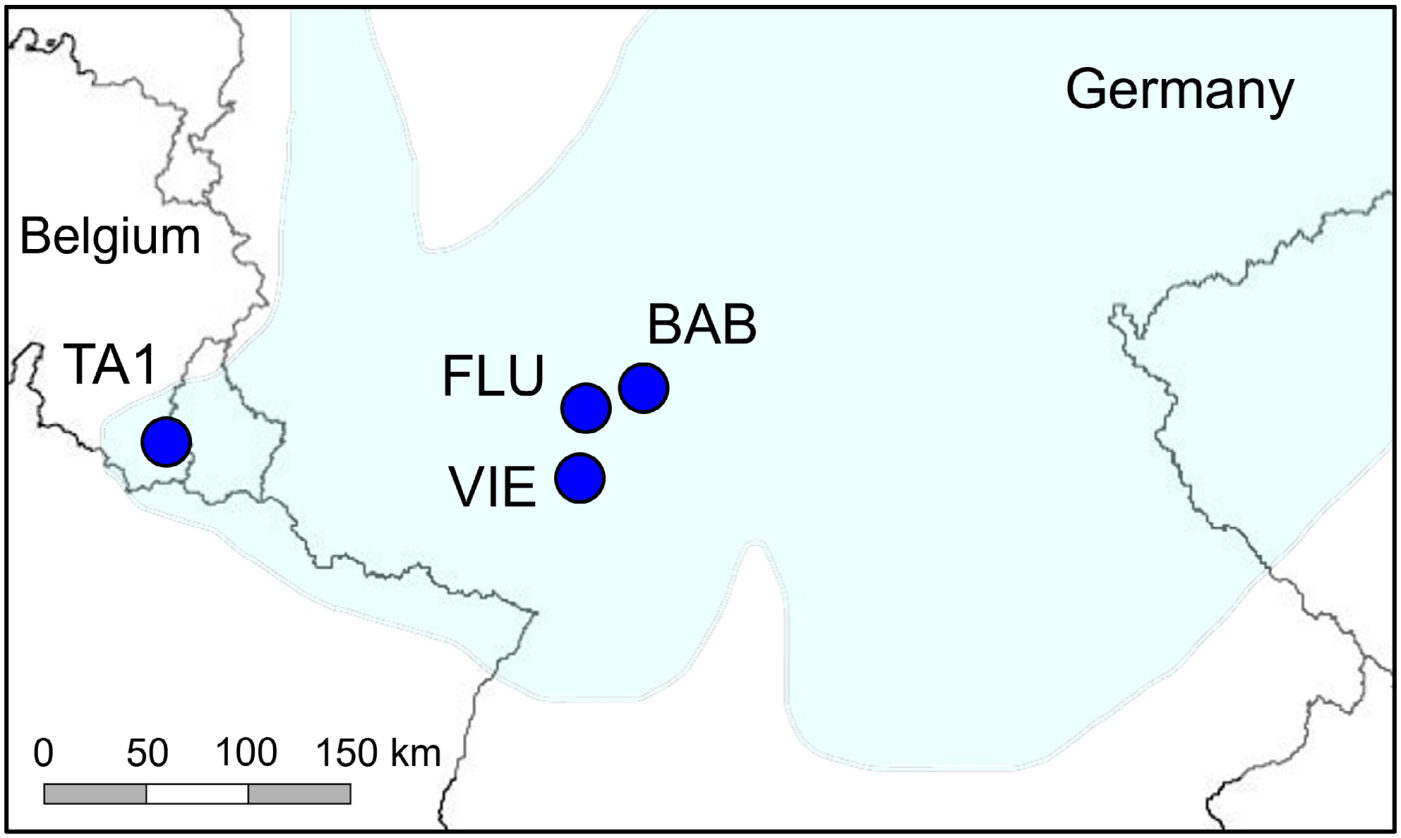
Location of the four studied populations of *Helichrysum arenarium* in Belgium (TA1) and in Germany (BAB, FLU and VIE). The colored area represents the historical distribution of the species (from Bettinger et al., 2013; Delvosalle, 2009; Pladias, 2014–2023).

Four populations were studied: the only remaining Belgian population (TA1) and the three large German seed source populations (BAB, FLU and VIE) where seeds were collected in August 2013 (Table 1, Figure 3; Godefroid et al., 2016). A total of 1900–4200 seeds per population were collected so that they were representative of the adult population, that is, across the whole population, from a high number of maternal plants (ranging from 50–100 to 100–1000 per population; Godefroid et al., 2016), distant from each other to avoid sampling seeds from closely related individuals (Commander et al., 2018; ENSCONET, 2009). German populations were 203–233 km apart from TA1 and 29–53 km apart from each other. Fresh leaves were collected from 10 adult rosettes (ramets) in TA1 and from seed progeny (= transplants used for plant translocations; see Godefroid et al., 2016) in BAB, FLU, and VIE (283 individuals in total; Table 1). Leaves were dried in silica gel.

**TABLE 1:** Details and estimates of within‐population genetic variation based on 11 microsatellite loci for the four studied populations of *Helichrysum arenarium* from Belgium and Germany: geographic coordinates, flowering population size (flow. pop. size), sample size (*n*), number of multilocus genotypes (*G*), proportion of polymorphic loci (*P*, in %), allelic richness (*A*_[8]_), observed (*H*_o_) and expected (*H*_e_) heterozygosity, Wright's inbreeding coefficient (*F*_IS_), inbreeding coefficient corrected for null alleles (*F*_Isnull_: mean value and 95% highest posterior density intervals); IIM model, best fitting model in the IIM analysis, including inbreeding (inbr, *nfb*) or not (no inbr, *nb*).

Population	Locality	Latitude (N)	Longitude (E)	Flow. pop. size	*n*	*G*	*P*	*A*_[8]_	*H*_o_	*H*_e_	*F*_IS_	*F*_Isnull_	IMM model
Belgium TA1	Tattert	49°42'50″	5°44′25″	6	10	2	63.6	1.71	0.636	0.340	−0.877*		
Germany BAB	Babenhausen	49°56′51″	8°57′55″	10^5^	90	89	100.0	4.42	0.520	0.635	0.176*	0.026 (0.000–0.062)	No inbr.
FLU	Flugplatz	49°51′16″	8°35′37″	10^5^	34	34	100.0	4.78	0.487	0.647	0.245*	0.086 (0.000–0.219)	No inbr.
VIE	Viernheim	49°33′16″	8°32′33″	10^5^	149	148	100.0	4.77	0.529	0.670	0.200*	0.024 (0.000–0.054)	No inbr.
Mean							90.9	3.92	0.543	0.573	−0.064		
SD							18.2	1.48	0.065	0.156	0.543		

*Note*: Departure from Hardy–Weinberg expectations: ns not significant, *p < .05 after Bonferroni correction.

### 2.2 Transferability and new development of microsatellites in simplex

Genomic DNA was isolated from ca. 15 mg of dried leaf tissue of 10 individuals from TA1, BAB, FLU, and VIE populations using a CTAB method (Doyle & Doyle, 1990). The DNA concentration was quantified using Qubit™ 3 (Thermo Fisher Scientific) and the DNA was diluted to 10 ng/μL.

The transferability of 12 microsatellite markers (HiUP‐13, HiUP‐18, HiUP‐22, HiUP‐24, HiUP‐01, HiUP‐02, HiUP‐04, HiUP‐05, HiUP‐08, HiUP‐15, HiUP‐16, and HiUP‐19) developed for *H. italicum* (Baruca Arbeiter et al., 2021) was tested on four *H. arenarium* samples from BAB (accession numbers Har612, Har632), FLU (Har675), and VIE (Har727). The DNA extracts were deposited in the genetic laboratory of the University of Primorska (Slovenia). The amplification of SSRs was performed in a final reaction volume of 12.5 μL containing 0.2 μM of each locus specific primer with one of the primers in pair that was elongated for M13(‐21) universal sequence (Schuelke, 2000), 0.25 μM of universal primer M13(‐21) labeled with 6‐FAM, VIC, PET, or NED (Applied Biosystems), 1× supplied AllTaq PCR Buffer (Qiagen), 1× supplied Q‐Solution (Qiagen), 2 mM MgCl2 (Qiagen), dNTP‐Mix (0.2 mM of each dNTP) (Qiagen), 1.25 unit of AllTaq DNA polymerase (Qiagen), and 40 ng of template DNA. The conditions of the two‐step PCR were as follows: initial denaturation at 94°C for 5 min, followed by 30 cycles at 94°C for 30 s, 45 s at the annealing temperature of 56°C, and the extension at 72°C for 45 s. The second step of amplification passed through 8 cycles of 30 s at 94°C, 45 s at the annealing temperature of 53°C, 45 s elongation at 72°C, and a final extension step at 72°C for 8 min (Schuelke, 2000). Twelve primer pairs showed successful amplification and polymorphism (Table S1). Separation of amplified microsatellites was performed on a SeqStudio™ Genetic Analyzer (Applied Biosystems) using GeneScan™ 500 LIZ (Applied Biosystems) as a size standard. Data were analyzed using GeneMapper v.4.1 (Applied Biosystems).

Two additional primer pairs (Ha‐ATC1 and Ha‐GAT1; Table 2) were developed using the same method as described in Van Rossum and Raspé (2018), that is, shotgun library preparation and pyrosequencing at Macrogen (Seoul, Korea), using one individual from BAB, and tested on 10 individuals from TA1, BAB, FLU, and VIE populations. The obtained sequence reads from pyrosequencing (64,752 reads of which a total of 2637 microsatellites of at least 5 repeats of di‐, tri‐ or tetranucleotide motifs were found) were submitted to the NCBI Sequence Read Archive (SRA) database under the accession number PRJNA1003501.

**TABLE 2:** Characteristics of the 11 microsatellite loci used in two multiplexes (A and B): fluorescent dye, primer amount used in the multiplex PCR (pmol), number of alleles (*An*), effective number of alleles (*Ae*), observed (*H*_o_) and expected (*H*_e_) heterozygosity, Fixation index (*F*), and null allele frequency (with its 95% highest posterior density interval).

Locus name	Multiplex	Dye	Allele size range (bp)	Primer amount (pmol)	*An*	*Ae*	*H*_o_	*H*_e_	*F*	Null allele frequency
HiUP−02	A	6‐FAM	139–159	0.2	7	3.5	0.739	0.712	−0.040ns	0.011 (0.000–0.028)
HiUP‐18	A	VIC	179–276	0.24	23	6.9	0.553	0.857	0.354*	0.183 (0.149–0.218)
HiUP‐01	A	NED	223–241	0.2	6	1.5	0.350	0.342	−0.025ns	0.018 (0.000–0.045)
HiUP‐16	A	PET	140–166	0.2	8	4.9	0.527	0.796	0.336*	0.195 (0.157–0.233)
HiUP‐05	A	PET	230–259	0.8	9	5.4	0.679	0.817	0.167*	0.091 (0.048–0.132)
HiUP‐13A	B	6‐FAM	137–139	0.2	3	1.3	0.131	0.208	0.371*	0.105 (0.058–0.159)
HiUP‐13B	B	6‐FAM	144–153	0.2	4	2.5	0.534	0.594	0.100ns	0.039 (0.000–0.073)
Ha‐GAT1	B	6‐FAM	176–197	0.2	7	4.0	0.713	0.749	0.046ns	0.031 (0.000–0.060)
HiUP‐19	B	6‐FAM	260–278	0.2	5	2.7	0.214	0.630	0.659*	0.416 (0.372–0.463)
Ha‐ATC1	B	6‐FAM	348–453	0.2	31	6.3	0.726	0.844	0.139*	0.090 (0.057–0.122)
HiUP‐08	B	PET	273–336	0.2	20	6.4	0.601	0.846	0.288*	0.138 (0.105–0.172)

*Note*: Departure from Hardy–Weinberg expectations: ns not significant, *p < .05 after Bonferroni correction.

### 2.3 Microsatellite multiplexing

For the 273 remaining samples, total genomic DNA was extracted from ca. 10 to 15 mg of dried leaf material that was finely grinded with ceramic beads in FastPrep‐24™ grinder (MP) with the NucleoMag 96 Plant kit (Macherey Nagel, Duren, Germany) according to the manufacturer's recommendations. We estimated the concentration of genomic DNA in the extracts using the Qubit Quantitation Platform (Invitrogen).

The 14 primer pairs were labeled with fluorescent dye (6‐FAM, VIC, NED, or PET; Di‐repeat + tail Applied Biosystems) to develop multiplexes using Multiplex Manager v.1.2 (Holleley & Geerts, 2009). From the 14 primer pairs tested with three multiplexes, one showed multiple peaks (HiUP‐24) and three did not amplify (HiUP‐22, HiUP‐04, HiUP‐15) so that 10 primer pairs (with HiUP‐13 consisting of two loci) were pooled into two multiplexes (Table 2). PCR amplifications were performed in 10 μL reactions containing 20 ng of template DNA, 1× Qiagen multiplex PCR master Mix, and 1× primer mix (0.2 μM of each primer, except 2.4 μM for HiUP‐18 and 8 μM for HiUP‐05). The PCR cycling consisted of an initial denaturation at 95°C for 15 min, followed by 30 cycles: denaturation at 95°C for 30 s, annealing at 55°C for 45 s and extension at 72°C for 1 min, and a final extension at 60°C for 30 min. Each PCR product was diluted with dH_2_O (1:100 for multiplex A and 1:50 for multiplex B) and mixed with Hi‐Di™ Formamide (Life Technologies, USA) and ® 500 dye Size Standard labeled with DY‐632 (Eurogentec). Fragments were separated using an ABI 3130XL DNA capillary sequencer (Applied Biosystems). Alleles were scored using GeneMapper v.5 (Applied Biosystems) and Geneious Prime (Biomatters).

### 2.4 Data analyses of the multiplexed loci

To verify the independence of the loci, a test for genotypic disequilibrium was performed between pairs of loci with sequential Bonferroni‐type correction on all samples using FSTAT v.2.9.4 (Goudet, 2003). For each locus, we calculated the number of alleles per locus (*An*), the effective number of alleles (*Ae*), observed (*H*_o_) and expected (*H*_e_) heterozygosity, and the Fixation index (*F*) over all populations using GenAlEx v.6.5 (Peakall & Smouse, 2012). Estimates of genetic variation over all loci were calculated for each population (for ramets for TA1) using GenAlEx and FSTAT: allelic richness (*A*_[*N*]_) for a fixed sample size (*N* = 8), observed (*H*_o_) and expected (*H*_e_) heterozygosity, and Wright's inbreeding coefficient (*F*_IS_), corrected for a small sample size. Deviations from Hardy–Weinberg expectations were tested for each locus and for each population over all loci by randomization tests and sequential Bonferroni‐type correction using FSTAT.

Using INEST v.2.2 (Chybicki & Burczyk, 2009), we estimated null allele frequencies for each locus and mean inbreeding coefficient values (*F*_ISnull_) for each population after adjusting allele frequencies with their 95% highest posterior density intervals (HPDI). Applying an IIM approach with 5 × 10^5^ Markov Chain Monte Carlo iterations, of which the first 5 × 10^4^ were discarded as burn‐in phase, we tested a full model (*nfb*, including null alleles, inbreeding and genotyping failures) and a model (*nb*) with no inbreeding. The best fitting model was indicated by the lowest value of the deviation information criterion (DIC). Clonal propagation in TA1 was estimated by calculating the probability (*p*_se_) of identical multilocus genotypes to be putative clones using GenAlEx. Clonality was not investigated in the German populations because genetic analyses were conducted on seed progeny. The HiUP‐05 locus that showed missing data for two individuals (and the same genotype for the other samples) was excluded from the analyses. The number of distinct multilocus genotypes within each German population was estimated by computing pairwise relationship coefficients between individuals according to Li et al. (1993), using SPAGeDi v.1.5d (Hardy & Vekemans, 2002). We assumed that two multilocus genotypes were identical between two individuals when the pairwise relationship coefficient was 1. Differences in *A*_[8]_, *H*_o_, and *H*_e_ values between populations were tested by performing pairwise Wilcoxon matched pairs tests by locus using STATISTICA v.12 (Dell Inc.).

Genetic structure among populations was investigated by calculating pairwise *F*_ST_ values between populations according to Weir and Cockerham (1984). Their significance was tested by randomization tests using FSTAT and Bonferroni correction. To take the presence of null alleles into account, pairwise *F*_ST_ values were also calculated with the *ENA* correction using FreeNa (Chapuis & Estoup, 2007). Bootstrap resampling over loci using 10,000 replicates were computed using FreeNa to obtain 95% confidence intervals. Additionally, population structure was also inferred using STRUCTURE v.2.3.4 (Pritchard et al., 2000). Analyses were performed after downloading the genotypic data on Galaxy web platform, using the public server at UseGalaxy.be (The Galaxy Community, 2022). This Bayesian clustering method identifies the number of clusters (*K*) of distinct gene pools that differ by a set of allele frequencies at each locus. Analyses were carried out for *K* = 1–6 clusters (10 independent runs), using an admixture ancestry model with correlated allele frequencies, run length of burn‐in period of 10^6^ iterations, and 2 × 10^6^ Markov Chain Monte Carlo replications. Null alleles were treated as recessive, and genotypes with missing data in the seven loci where null alleles were detected were considered as homozygous for null alleles (Falush et al., 2007). The most likely number of *K* clusters was inferred based on the ad hoc statistic Delta*K* and the highest likelihood value as described in Evanno et al. (2005), after running STRUCTURE HARVESTER (Earl & vonHoldt, 2012). For each *K*, the independent run with the highest likelihood value was visualized on barplots using DISTRUCT v.1.1 (Rosenberg, 2004). A principal coordinate analysis (PCoA) based on a standardized genetic distance matrix (Smouse & Peakall, 1999) was also performed using GenAlEx.

## 3 RESULTS

The 11 loci were effectively amplified for all the study populations. Single‐locus genotypes could not be determined for two Belgian samples at one locus and for 75 German samples at one to four loci. Genotypic disequilibrium was significant for only one of the 55 pairs after sequential Bonferroni correction (*p* = .00023) so that these loci could be considered as mostly independent. Three to 31 alleles were scored in the 11 loci, for 123 alleles in total. At locus level, *H*_o_ and *H*_e_ varied from 0.131 to 0.739 and from 0.208 to 0.857, respectively (Table 2). Seven loci significantly deviated from Hardy–Weinberg expectations, with a deficit in heterozygotes (positive *F* values). There was also indication for the presence of a null allele for those loci (95% HDPI values differing from 0), with five loci showing with a mean proportion higher than 0.100, up to 0.416 for HiUP‐19 (Table 2). The IIM analysis for the overall dataset indicated that the significantly positive *F* values could be attributed to null allele presence rather than to inbreeding (lowest DIC for the *nb* model).

At population level, allelic richness varied from 1.71 to 4.77 and *H*_o_ and *H*_e_ from 0.487 to 0.636 and from 0.340 to 0.670, respectively (Table 1). A total of 42 private alleles were found: 2 in TA1, 9 in BAB and FLU, and 22 in VIE. The three German populations (BAB, FLU, VIE) showed significantly higher values of allelic richness and *H*_e_ than those of TA1, the only small population remaining in Belgium (pairwise Wilcoxon matched pairs tests, *Z* = 2.93, *p* < .01; Table 1, Table S2). The other tests (between German populations and for *H*_o_) were not significant (*p* > .05, Table S2). Only two distinct multilocus genotypes (genets) were found in TA1, and nine of the 10 sampled rosettes corresponded to one genet and could be considered as putative clones (*p*_se_ < 0.05). Only a few individuals had the same multilocus genotype within each German population, and a total of 264 distinct multilocus genotypes was found for the 273 individuals. Mean *F*_IS_ value was significantly negative (excess in heterozygotes) for TA1, and positive for the three German populations, but those were not significant anymore after correction for null allele frequency (lowest HPDI values of *F*_ISnull_ = 0; lowest DIC for the *nb* model) (Table 1).

**FIGURE 4:**
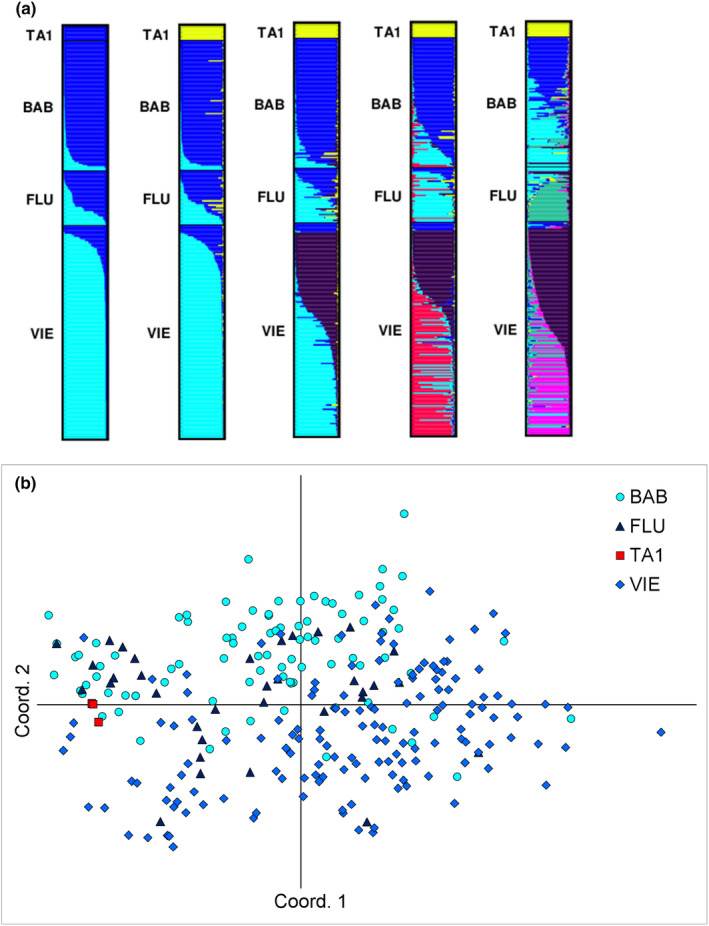
(a) Results of Bayesian clustering (modal *K* = 2–6). In the barplot, each individual is represented by a horizontal bar, showing the membership (*Q*) to each of the modal clusters; (b) Principal Coordinate Analysis (PCoA) plot for four populations of *Helichrysum arenarium* from Belgium (TA1, only consisting of two different multilocus genotypes) and Germany (BAB, FLU and VIE). Axes 1 and 2 explained 15.3% of the total variation.

Genetic differentiation between German populations was low (*F*_ST_ values ranging 0.035–0.047 and 0.033–0.045 with the *ENA* correction), seeming related with geographic distances (Table S3), and high between TA1 and the three German populations (*F*_ST_ values ranging 0.186–0.206 and 0.195–0.213 with the *ENA* correction). All *F*_ST_ values were significant (*p* < .05, Table S3). The STRUCTURE analysis gave the best modal clusters at *K* = 2, and also at *K* = 5 according to Evanno's method (Figure S1), and revealed some genetic structure among German populations (Figure 4a): most individuals of BAB and VIE were assigned to distinct clusters at *K* = 2, with 90%–86% of the individuals ascribed to one cluster with *Q* membership ≥80%, respectively. Yet, 3%–5% of the individuals were ascribed to the other cluster with *Q* membership ≥80%, respectively (Table S4). Individuals of FLU were assigned to both clusters, suggesting a possible spatial substructure (Figure 4a). TA1 clustered apart in the STRUCTURE analysis from *K* = 3 (Figure 4a; Table S4), but genotypic patterns still remained within the range of variation of the German populations in the PCoA plot (Figure 4b).

## 4 DISCUSSION

### 4.1 Genetic diversity assessment

Assessing the genetic status of fragmented remnant populations of (locally) critically endangered species is important for achieving appropriate restoration actions, especially when species are characterized by a self‐compatible breeding system and/or by clonal propagation ability (e.g., Gargiulo et al., 2023; Tierney et al., 2020; Van Rossum et al., 2021). The last small natural population from Belgium (TA1) of *H. arenarium* showed high clonality, with only two distinct adult genets found in the sampled rosettes, however, still retaining some genetic diversity as shown by a *H*_o_ value similar to those found in the large German populations (estimated on progeny obtained from seed bulks, that is, the transplants that will be used for plant translocation). The high heterozygosity level of the two remaining genets suggests that the remaining plants of this long‐lived clonal perennial are old adults, possibly recruited before population demographic decline (Bittebiere et al., 2020; Van Rossum et al., 2022; Van Rossum & Raspé, 2018). Heterosis might have favored the survival of heterozygous individuals (Barmentlo et al., 2018) as reported in relictual populations of *A. montana* (Luijten et al., 2002). This population can be considered as nonviable given its very small size, confirming the need for a reinforcement. High clonality resulting in a low number of genets was also found in small populations of the endangered *Arnica montana* L. (Van Rossum & Raspé, 2018), *Persoonia hindii* P.H. Weston & L.A.S. Johnson (Tierney et al., 2020), and *Hibbertia spanantha* Hellmut R. Toelken & A.F. Rob. (Doyle et al., 2023).

Using large—genetically diverse—populations as seed sources for plant translocations is recommended to optimize population establishment and evolutionary resilience, and to avoid inbreeding issues in the reintroduced gene pool (Commander et al., 2018; Menges, 2008; Schäfer et al., 2020). As expected, high genetic diversity (*H*_e_ = 0.64–0.67) was found in the three very large German populations. Similar values of genetic diversity based on microsatellite loci have been reported for large populations of other species concerned by restoration involving plant translocations, such as *A. montana* (*H*_e_ = 0.55–0.59; Van Rossum & Raspé, 2018) and *Cypripedium calceolus* L. (*H*_e_ = 0.51–0.68; Gargiulo et al., 2021).

### 4.2 Genetic differentiation patterns

There was high genetic differentiation between Belgian and German populations, but the two Belgian genotypes only showed two private alleles and remained within the range of genetic variation of German populations so that this pattern likely rather resulted from genetic drift effects and small sample size. Whether Belgian and German populations are genetically similar might be verified by genotyping herbarium specimens from former Belgian populations existing before species decline (Rosche et al., 2022). Some, however weak, genetic differentiation among German populations was detected, suggesting an isolation‐by‐distance pattern (which, however, cannot be tested given the small number of populations).

### 4.3 Low inbreeding in future transplants

No significant inbreeding was detected in the seed progeny (future transplants for translocations) of the large German populations when taking null allele frequency into account, suggesting random mating and extensive pollen and seed dispersal. Nevertheless, the genetic data remain compatible with low inbreeding coefficients in each population, with *F*_ISnull_ values up to 0.024–0.086 (Table 1). Flowers of *H. arenarium* are considered as important nectar and pollen resources for many insects, in particular for solitary bees, wasps, and flies (e.g., Beil et al., 2008, 2014). Pollen dispersal patterns by these insects are often leptokurtic‐shaped (fat‐tailed), with most allogamous pollination events occurring among neighbors, so at relatively short distances, and with a few long‐distance pollen transfers (e.g., Hardy et al., 2004; Van Rossum et al., 2011; Zurbuchen et al., 2010). The presence of a hairy pappus on achenes of *H. arenarium* (Figure 2) indicates wind‐dispersal ability (Klotz et al., 2002; Pljevljakušić et al., 2018). Despite the fact that dispersal distances can be increased with a pappus, wind‐generated seed rains also usually show fat‐tailed distributions (Nathan et al., 2011; Nathan & Muller‐Landau, 2000). Therefore, positive inbreeding coefficient values might have been expected, related to within‐population spatial structuring of the genetic variation (Wahlund effect), with mating among relatives, resulting in biparental inbreeding (Monks et al., 2021; Thomas et al., 2021; Vekemans & Hardy, 2004). The source sites in Germany were extensively grazed by livestock, in particular sheep in BAB, and sheep and donkeys in FLU (mown until 1992, sheep grazing since 1999) and in VIE (Eichberg et al., 2007; Storm et al., 2019; S. Godefroid, pers. comm.). Extensive grazing by livestock as management practice can potentially promote long‐distance seed dispersal by endo‐ or epizoochory (seeds carried in fur or under hooves), reducing spatial genetic structure and inbreeding in the populations (Lehmair et al., 2020; Plue et al., 2019; Rico & Wagner, 2016). However, epi‐ and endozoochory by sheep has been investigated for sand grassland species in the FLU study site, and for *H. arenarium*, no seeds were found in the sheep coat nor germinated from sheep dung (Eichberg et al., 2007; Wessels, 2007; Wessels et al., 2008). The Bayesian clustering also suggests some genetic substructure in FLU (Figure 4). In this site, *H. arenarium* occurs in two spatially distinct habitats, an open xeric sand calcareous grassland vegetation (*Koelerion glaucae*) in the eastern part, and a seminatural more productive dry grassland (*Armerio elongatae*‐*Festucetum trachyphyllae*) in the central and western parts. FLU also served as a military airbase until 1992 so that the grasslands are fragmented by landing, road, and building infrastructures (Storm et al., 2019).

Finding nonsignificant inbreeding values is also surprising given the possibility of self‐pollination, as *H. arenarium* is characterized by a self‐compatible breeding system and extensive clonal propagation ability (Godefroid et al., 2016; Klotz et al., 2002; Knuth, 1908). Indeed, the combination of both traits may increase geitonogamous fertilization opportunities (Bittebiere et al., 2020; Vallejo‐Marín et al., 2010), possibly resulting in high inbreeding levels (Somme et al., 2014; Van Rossum & Le Pajolec, 2021). Moreover, homogamous florets simultaneously flower on many capitula, with stylar branches first erect then bending between the anthers (Knuth, 1908). Also, inflorescences are structured as false umbels of numerous capitula, forming landing pads for insects (Pljevljakušić et al., 2018; Figure 1). Those traits are expected to promote (possibly delayed) geitonogamous self‐pollination (Goodwillie et al., 2005; Goodwillie & Weber, 2018). In our study, however, assuming that the actual *F*_IS_ ≤ 0.054 (based on *F*_ISnull_ value of the best sampled population VIE; Table 1) implies that the selfing rate (*s*) is ≤0.1, given that the equilibrium inbreeding expected under selfing equals *F*_IS_ = *s*/(2 − *s*) (Weir, 1996).

Various, possibly interacting mechanisms are known to prevent the occurrence of selfed individuals in self‐compatible plant species. First, intermingling of clones from different genets may contribute to reduce selfing rates (Albert et al., 2008). Second, *H. arenarium* has been reported as gynomonoecious, with external florets that can be female, starting to flower before the central florets of the capitula (Klotz et al., 2002; Pljevljakušić et al., 2018), which may promote allogamous pollination (Mamut et al., 2014). Third, postpollination processes may occur, such as delayed selfing through delayed germination of self‐pollen tubes, competition of self‐pollen with outcross pollen, and competition of selfed ovules with outcrossed ovules (e.g., for resource provisioning) (Charlesworth, 2006; Goodwillie & Weber, 2018). Finally, early inbreeding depression may lead to abortion of self‐fertilized embryos or to nonviable inbred seedlings (Colling et al., 2004; Goodwillie et al., 2005; Luijten et al., 2000). As a result, inbreeding levels in adults may remain low (Van Rossum et al., 2006; Yang & Hodges, 2010). Partial self‐incompatibility may also occur: transient self‐incompatibility prevents selfing early in anthesis, and cryptic self‐incompatibility only allows for selfing when cross‐pollen is absent (Goodwillie & Weber, 2018). Seed abortion and juvenile mortality (8.5%) in good cultivation conditions have been found for seeds collected in translocated populations of *H. arenarium* from southern Belgium (B. Barigand, unpubl. data; F. Van Rossum et al., unpubl. data), suggesting possible inbreeding depression or partial self‐incompatibility mechanisms. Whether selective processes might act against selfing in *H. arenarium* certainly deserves to be further investigated, for example, by assessing proportion of female florets in capitula, by floral bagging and hand‐pollination experiments, and by sibship reconstruction and paternity analyses of seed embryos (before germination) and seed progeny arrays (after germination), allowing among others to estimate selfing rates (e.g., Charlesworth, 2006; Chybicki, 2018; Goodwillie & Weber, 2018; Hufford & Hamrick, 2003; Van Rossum, 2023). However, given the tiny size of the seeds, isolating embryos for DNA extraction might not be possible.

## 5 CONCLUSION

*Helichrysum arenarium* is on the brink of extinction in southern Belgium as well as in the surrounding regions, that is, in eastern France, Grand Duchy of Luxembourg and The Netherlands (e.g., Godefroid et al., 2016). Our genetic study showed evidence of the need for plant translocations for reinforcement of the last remaining populations of *H. arenarium* in Belgium. For species recovery in the region, reintroductions should also be implemented, using nonlocal, possibly large, seed source populations (Commander et al., 2018). The transplants obtained from seeds sampled from the three populations from western Germany, the geographically closest large populations that occurred in similar ecological conditions, constitute a highly diverse, noninbred gene pool, and are thus of high genetic quality for plant translocations. Given that our results did not allow for apprehending regional genetic diversity of *H. arenarium* in southern Belgium (due to population extirpation), and in order to optimize population evolutionary genetic resilience and adaptive potential to the translocation sites in a context of possibly changing environments, mixing the three seed source populations should be considered (Broadhurst et al., 2023; Monks et al., 2021). Further investigating seed and pollen dispersal patterns to identify the mating processes (and in particular selfing and admixture between sources) and the dispersal vectors responsible for genetic structure can contribute to understand population genetic dynamics during translocated population establishment.

## AUTHOR CONTRIBUTIONS

**Fabienne Van Rossum:** Conceptualization (lead); formal analysis (lead); investigation (supporting); visualization (lead); writing – original draft (lead). **Cécile Godé:** Investigation (lead); methodology (equal); writing – original draft (equal). **Alenka Baruca Arbeiter:** Investigation (equal); methodology (equal); writing – original draft (equal). **Olivier Raspé:** Investigation (equal); methodology (equal); writing – review and editing (equal). **Melike Simsek:** Investigation (equal); writing – review and editing (equal). **Benjamin Barigand:** Investigation (equal); writing – review and editing (equal). **Olivier J. Hardy:** Conceptualization (equal); formal analysis (equal); writing – original draft (equal). **Dunja Bandelj:** Investigation (equal); methodology (equal); writing – original draft (equal).

## CONFLICT OF INTEREST STATEMENT

The authors declare no conflicts of interest.

## Supporting information


Data S1

## Data Availability

DNA sequence information have been deposited in Genbank (accessions MT992215‐MT992238) and in NCBI SRA (accession PRJNA1003501). The dataset presented in this study can be found in online Zenodo repository at https://doi.org/10.5281/zenodo.8226065.
