# Supplementary material for: Genetic diversity assessment of Helichrysum arenarium (Asteraceae) for the genetic restoration of declining populations
Source: Ecol Evol. 2024 Feb 15;14(2):e10953. doi: 10.1002/ece3.10953 (PMC10869947; doi:10.1002/ece3.10953)
Supplement: Supplementary file 1 — Data S1 [file ECE3-14-e10953-s001.docx]

**Genetic diversity assessment of *Helichrysum arenarium* (Asteraceae) for the genetic restoration of declining populations - Supplementary Material**

**TABLE S1** Characteristics of the 14 developed microsatellite loci.

| Locus name | GenBank Accession No. | Primer sequence (5’–3’) | Repeat motif | Size range (bp) |
| --- | --- | --- | --- | --- |
| Ha-GAT1 | PRJNA1003501 | F: CCTGCGTAATCGCGGGTC | (GAT)_12_ | 176-194 |
|  |  | R: GTTTCTCTTCCGGCCGTTG |  |  |
| Ha-ATC1 | PRJNA1003501 | F: TTAATTGTCCATCAACGTACCC | (ATC)_13_ | 364-385 |
|  |  | R: GCTTCGAAATTTATGCAATATGCTG |  |  |
| HiUP-01 | MT992215 | F: CAACATCAAACATGGCATCTG* | (AC)_10_ | 242-260 |
|  |  | R: ATGTAATCCGCCCAATGCT |  |  |
| HiUP-02 | MT992216 | F: TTGCTTTGTATTGCTGTCTATCC* | (AC)_10_ | 154-179 |
|  |  | R: GGAAACGTCCTGCAAGATTA |  |  |
| HiUP-04 | MT992218 | F: TCTATACAAACCAGTCCTTCCC* | (AC)_10_ | 139-157 |
|  |  | R: TGAGCACAGCTAGTTTCTCCC |  |  |
| HiUP-05 | MT992219 | F: TGGCGAATTCTAAGCCTTGT* | (AC)_14_ | 245-279 |
|  |  | R: AATCATGATGAACAGCTGACG |  |  |
| HiUP-08 | MT992222 | F: GGAAGTGCAAATAGATGAACG* | (AC)_10_ | 270-310 |
|  |  | R: TGGTTGATTATGGATGTTGCTT |  |  |
| HiUP-13 | MT992227 | F: CCAAGTTGATGATCTCCAATGA* | (ATC)_8_ | 156-178 |
|  |  | R: TATCCATGGCTTTCTACCCA |  |  |
| HiUP-15 | MT992229 | F: TGACCGCTCGAATATTCTTTG* | (AAT)_7_ | 143-153 |
|  |  | R: AACCAAGTACAACGCCATTT |  |  |
| HiUP-16 | MT992230 | F: GGGCCTGTAATGAAGGTGTT* | (AGC)_7_ | 155-186 |
|  |  | R: TATTGCGCGATCCTGATTCT |  |  |
| HiUP-18 | MT992232 | F: CCACTAACCATCAACCCTGG* | (ACT)_7_ | 194-217 |
|  |  | R: ATGATGATCCATCGAACTGTAATC |  |  |
| HiUP-19 | MT992233 | F: AACCGTTGCATCATCACAGA* | (ATC)_10_ | 260-302 |
|  |  | R: TACCAAGGTACTGCGGAACA |  |  |
| HiUP-22 | MT992236 | F: TTGCTCACACCTGGATTCG* | (AAT)_7_ | 252-293 |
|  |  | R: CGTATTGATTGCAATGCCTTT |  |  |
| HiUP-24 | MT992238 | F: TTGGAGTACAAGGTTGGGAA* | (AAT)_9_ | 264-284 |
|  |  | R: CCGGTACATTAACACCCACC |  |  |

* Primer has been elongated for M13 (-21) 18 bp sequence (5'-TGTAAAACGACGGCCAGT-3')

**TABLE S2** Wilcoxon pairs matched tests (*Z* value) comparing four populations of *Helichrysum arenarium* from Belgium and Germany.

|  |  | *A*_[8]_ |  |  | *H*_o_ |  |  | *H*_e_ |  |
| --- | --- | --- | --- | --- | --- | --- | --- | --- | --- |
| Population pair | | *Z* | *P* |  | *Z* | *P* |  | *Z* | *P* |
| TA1 | BAB | 2.93 | ** |  | 1.16 | ns |  | 2.93 | ** |
| TA1 | FLU | 2.93 | ** |  | 1.24 | ns |  | 2.93 | ** |
| TA1 | VIE | 2.93 | ** |  | 1.07 | ns |  | 2.93 | ** |
| BAB | FLU | 1.69 | ns |  | 0.36 | ns |  | 0.53 | ns |
| BAB | VIE | 1.51 | ns |  | 0.00 | ns |  | 1.33 | ns |
| FLU | VIE | 0.18 | ns |  | 0.44 | ns |  | 0.98 | ns |

Abbreviations: *A*_[8]_ allelic richness, *H*_o_ observed heterozygosity, *H*_e_ expected heterozygosity; ns not significant; ** *P* < 0.01.

**TABLE S3** Pairwise genetic differentiation [*F*_ST_ values without (upper half of the matrix) and with the ENA correction (lower half of the matrix)] and geographic distances (in km) between four populations of *Helichrysum arenarium* from Belgium and Germany.

|  |  | *F*_ST_ |  |  |  | Geographic distance (km) | | |
| --- | --- | --- | --- | --- | --- | --- | --- | --- |
|  | TA1 | BAB | FLU | VIE |  | BAB | FLU | VIE |
| TA1 |  | 0.186 | 0.191 | 0.206 |  | 233 | 205 | 203 |
| BAB | 0.195 |  | 0.035 | 0.047 |  |  | 29 | 53 |
| FLU | 0.208 | 0.033 |  | 0.038 |  |  |  | 34 |
| VIE | 0.213 | 0.045 | 0.035 |  |  |  |  |  |

All values are significant (*P* < 0.05 after Bonferroni correction or lowest 95% confidence interval > 0 with the ENA correction).

**TABLE S4** Proportion of individuals assigned to a particular cluster with a membership (*Q*) ≥ 80 % (modal *K* = 2 to 6).

|  | *K* = 2 | |  | *K* = 3 | | |  | *K* = 4 | | | |
| --- | --- | --- | --- | --- | --- | --- | --- | --- | --- | --- | --- |
| Population | *Q*_1_ | *Q*_2_ |  | *Q*_1_ | *Q*_2_ | *Q*_3_ |  | *Q*_1_ | *Q*_2_ | *Q*_3_ | *Q*_4_ |
| BAB | 0.03 | 0.90 |  | 0.92 | 0.03 | 0.00 |  | 0.02 | 0.81 | 0.00 | 0.00 |
| FLU | 0.18 | 0.45 |  | 0.50 | 0.18 | 0.00 |  | 0.29 | 0.18 | 0.00 | 0.00 |
| TA1 | 0.00 | 1.00 |  | 0.00 | 0.00 | 1.00 |  | 0.00 | 0.00 | 0.00 | 1.00 |
| VIE | 0.86 | 0.05 |  | 0.05 | 0.83 | 0.00 |  | 0.41 | 0.05 | 0.33 | 0.00 |

|  |  |  | *K* = 5 |  |  |  | *K* = 6 | | | | | |
| --- | --- | --- | --- | --- | --- | --- | --- | --- | --- | --- | --- | --- |
| Population | *Q*_1_ | *Q*_2_ | *Q*_3_ | *Q*_4_ | *Q*_5_ |  | *Q*_1_ | *Q*_2_ | *Q*_3_ | *Q*_4_ | *Q*_5_ | *Q*_6_ |
| BAB | 0.02 | 0.00 | 0.66 | 0.00 | 0.04 |  | 0.00 | 0.01 | 0.31 | 0.16 | 0.00 | 0.01 |
| FLU | 0.05 | 0.00 | 0.08 | 0.00 | 0.55 |  | 0.00 | 0.45 | 0.00 | 0.11 | 0.00 | 0.05 |
| TA1 | 0.00 | 1.00 | 0.00 | 0.00 | 0.00 |  | 1.00 | 0.00 | 0.00 | 0.00 | 0.00 | 0.00 |
| VIE | 0.34 | 0.00 | 0.04 | 0.26 | 0.05 |  | 0.00 | 0.05 | 0.01 | 0.03 | 0.24 | 0.29 |

**
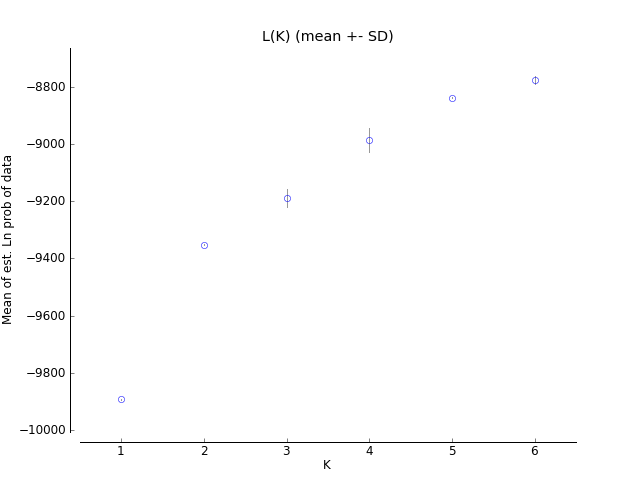

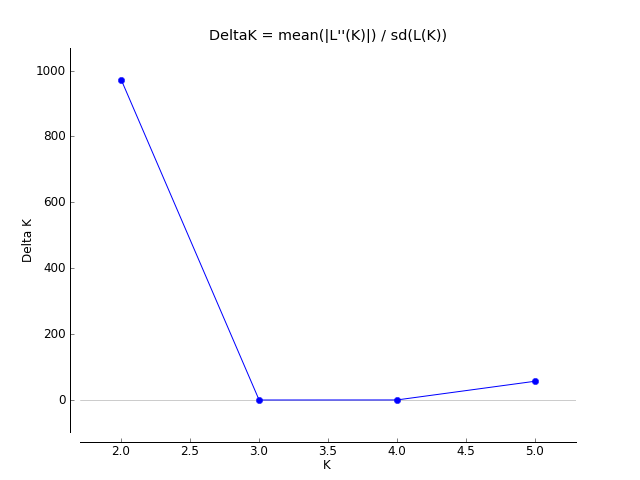
**

**FIGURE S1** Plots of LnP(*K*) and Delta *K* in function of *K* for four populations of *Helichrysum arenarium* from Belgium and Gemany*.*
